# Ovalbumin-related Protein X Is a Heparin-binding Ov-Serpin Exhibiting Antimicrobial Activities*[Fn FN2]

**DOI:** 10.1074/jbc.M113.469759

**Published:** 2013-04-24

**Authors:** Sophie Réhault-Godbert, Valérie Labas, Emmanuelle Helloin, Virginie Hervé-Grépinet, Cindy Slugocki, Magali Berges, Marie-Christine Bourin, Aurélien Brionne, Jean-Claude Poirier, Joël Gautron, Franck Coste, Yves Nys

**Affiliations:** From the ‡ Institut National de la Recherche Agronomique, UR83 Recherches Avicoles, Fonction et Régulation des Protéines de l'Œuf, F-37380 Nouzilly, France,; § Institut National de la Recherche Agronomique, UMR85 Physiologie de la Reproduction et des Comportements, Plate-forme d'Analyse Intégrative des Biomarqueurs, Laboratoire de Spectrométrie de Masse et de séquençage, F-37380 Nouzilly, France,; ¶ Institut National de la Recherche Agronomique, ISP311, Institut National de la Recherche Agronomique, UMR1282, Infectiologie et Santé Publique, Centre International de Ressources Microbiennes-Bactéries Pathogènes, F-37380 Nouzilly, France,; ‖ Inserm UMR 1100, EA 6305 Centre d'Etude des Pathologies Respiratoires, F-37032 Tours, France,; ** Institut Technique de l'Aviculture, UR83 Recherches Avicoles, F-37380 Nouzilly, France, and; ‡‡ CNRS, UPR4301 Centre de Biophysique Moléculaire, Réparation de l'acide désoxyribonucléique: approches structurales et fonctionnelles, F-45071 Orléans, France

**Keywords:** Glycoprotein, Heparin Binding Protein, Innate Immunity, Protein Structure, Serpin, Antibacterial Proteins, Chicken Egg

## Abstract

Ovalbumin family contains three proteins with high sequence similarity: ovalbumin, ovalbumin-related protein Y (OVAY), and ovalbumin-related protein X (OVAX). Ovalbumin is the major egg white protein with still undefined function, whereas the biological activity of OVAX and OVAY has not yet been explored. Similar to ovalbumin and OVAY, OVAX belongs to the ovalbumin serine protease inhibitor family (ov-serpin). We show that OVAX is specifically expressed by the magnum tissue, which is responsible for egg white formation. OVAX is also the main heparin-binding protein of egg white. This glycoprotein with a predicted reactive site at Lys^367^-His^368^ is not able to inhibit trypsin, plasmin, or cathepsin G with or without heparin as a cofactor. Secondary structure of OVAX is similar to that of ovalbumin, but the three-dimensional model of OVAX reveals the presence of a cluster of exposed positive charges, which potentially explains the affinity of this ov-serpin for heparin, as opposed to ovalbumin. Interestingly, OVAX, unlike ovalbumin, displays antibacterial activities against both *Listeria monocytogenes* and *Salmonella enterica* sv. Enteritidis. These properties partly involve heparin-binding site(s) of the molecule as the presence of heparin reverses its anti-*Salmonella* but not its anti-*Listeria* potential. Altogether, these results suggest that OVAX and ovalbumin, although highly similar in sequence, have peculiar sequential and/or structural features that are likely to impact their respective biological functions.

## Teaser

**Background:** Ovalbumin-related protein X (OVAX) is an uncharacterized ovalbumin-serpin.

**Results:** This egg white-specific serpin lacks protease inhibitory activity, but unlike its ovalbumin homolog, OVAX exhibits antibacterial properties, partly through its heparin-binding site(s).

**Conclusion:** OVAX, a non-inhibitory serpin is a heparin-binding molecule with antibacterial activity.

**Significance:** OVAX participates in egg defense and constitutes a natural agent against *Listeria* and *Salmonella*.

## Introduction

The ovalbumin gene family in *Gallus gallus* is composed of a cluster of three genes, ovalbumin, ovalbumin-related protein Y (OVAY),^2^ and ovalbumin-related protein X (OVAX) genes located on chromosome 2 within a 40-kb region (1, 2). OVAY and OVAX genes share highly significant similarity in coding sequences with the ovalbumin gene, suggesting that they have arisen by duplication from a common ancestor gene (1, 2). All three genes are paralogs and have no orthologs in humans (3). The expression of these three genes in hen oviduct is under estrogen control, but their relative hormonal responsiveness and their resulting expression in chicken oviduct differs in the order of ovalbumin:OVAY:OVAX = 100:10:1 (4). There is, to date, no information related to the mechanisms underlying the differential hormone responsiveness of these highly-related genes (5, 6).

Ovalbumin belongs to the serine protease inhibitor (serpin) family of which members share a common tertiary structure. This conserved structure consists of three β-sheets, nine α-helices, and a solvent-exposed reactive center loop. The latter is composed by a flexible stretch of ∼17 residues, which interacts with target proteases. The proteolytic cleavage of this loop leads to either a kinetically trapped loop-inserted covalent complex between the protease and the serpin (inhibitory pathway) or to a cleaved serpin and a free protease (non-inhibitory or substrate pathway) (7). Ovalbumin is part of the ovalbumin-related serpin (ov-serpin) subgroup defined by short N and C termini compared with the archetype α_1_-antitrypsin and an absence of a classical secretory signal peptide (8, 9). Ovalbumin (SERPINB14) together with maspin/SERPINB5 is one of the few members of this family that is not a protease inhibitor. The biological activities of ovalbumin in hen egg are still not defined. Bioinformatical analysis of OVAY and OVAX revealed that both proteins are also ov-serpins, named SERPINB14B and SERPINB14C, respectively. The alignment of their respective reactive site loop with that of inhibitory ov-serpins indicated that both proteins have hinge regions that deviate less from the amino acid consensus sequence for inhibitory ov-serpins than that of non-inhibitory ovalbumin, and some have suggested that OVAY and OVAX may be inhibitory (10). The protein sequence of OVAX is still not fully defined: it appears as a predicted sequence related to OVAY in the NCBI database: predicted sequence, ovalbumin-related protein Y (*Gallus gallus*) (XP_418984.3), whereas only a fragment of the protein is available in the Uniprot database (OVALX_CHICK, P01013). Additionally, at present, there are no comprehensive studies exploring the biological activities of OVAX in egg. In this regard, to further investigate the function of OVAX, we first developed a purification process based on heparin-affinity chromatography. We analyzed the biochemical properties of OVAX (post-translational modifications, content in secondary structures, proteases inhibitory activities), whereas cloning of the corresponding mRNA combined to mass spectrometry allowed us to confirm/refine its predicted protein sequence. Moreover, through OVAX structure modeling, we elucidated a putative domain involved in binding of OVAX to heparin. Because some heparin-binding proteins have been reported to exhibit antimicrobial activity (11–14), we examined the antibacterial potential of OVAX against Gram-positive and Gram-negative bacterial strains.

## EXPERIMENTAL PROCEDURES

### 

#### 

##### Materials

Bovine trypsin, PNGase F from *Elizabethkingia meningosepticum* (EC 3.5.1.52), biotin-agarose, heparin from porcine intestinal mucosa, plasmin and S-free ovalbumin grade VII, and bovine serum albumin were from Sigma-Aldrich. Cathepsin G from human neutrophils (EC 3.4.21.20), α1-antichymotrypsin from human plasma, α1-antitrypsin from human, and plasma α2-antiplasmin were purchased from MP Biomedicals (Illkirch, France). Heparin-Sepharose beads were obtained from GE Healthcare. All other chemicals were analytical grade.

##### Real-time Quantitative-Polymerase Chain Reaction

Eight laying hens (ISA Brown, Hendrix Genetics, St-Brieuc, France) were bred at the experimental unit UE PEAT 609 according to the European legislation (86/609/EEC) and under the supervision of two authorized scientists S. Réhault-Godbert and J. Gautron (authorizations 7323 and 37-144, respectively). Hens were euthanized, and tissues were harvested from infundibulum, white isthmus, magnum and uterus, liver, kidney, and duodenum. Real-time Quantitative-Polymerase Chain Reaction was performed as described (15) using primers 5′-AAGACAGCACCAGGACACAGA-3′ (forward) and 5′-TTCTGGCAGATTGGGTATC-3′ (reverse) for ovalbumin (amplicon = 212 base pairs) and 5′-TCCGTGAACATCCACCTACTCT-3′ (forward) and 5′-GGCTTGGTCTGATGCTGTTT-3′ (reverse) for OVAX (amplicon = 198 base pairs). Ovalbumin and OVAX mRNA levels were corrected relative to ribosomal 18 S rRNA. The ratio value was calculated for each sample as “OVAX” or ovalbumin/18 S RNA. The log of the ratio was used for statistical analysis using one-way analysis of variance (StatView software, version 5, SAS Institute, Inc.).

##### Amplification of OVAX mRNA

The coding sequence of OVAX (XM_418984.3, CDS: 26 to 1219 bp) was amplified from magnum total RNAs. Primers used for amplification were 5′-ACGAGCCTCTTTGATGTTTTTCT-3′ and 5′-CCTCTCCTGTGGGTATGTCT-3′ for the forward and reverse primers. PCR reaction was achieved using the platinum pfx DNA polymerase kit (Invitrogen), PCR product was purified using Illusta GFX gel band purification kit (GE Healthcare) and sequenced (Beckman Coulter Genomics, Essex, United Kingdom).

##### Purification of OVAX

Egg whites were prepared as published previously (16), diluted 1:1 in 50 mm Tris-HCl, 300 mm NaCl, pH 7.4, and was incubated with heparin-Sepharose beads. The beads were washed with 50 mm Tris-HCl, 150 mm NaCl, pH 7.4. Elution of bound proteins was achieved with 50 mm Tris-HCl, 1 m NaCl, pH 7.4. Eluted fractions were concentrated (Ultracel-3K, Millipore, Molsheim, France) and injected on a Hiprep 16/60 Sephacryl S-100 High Resolution (GE Healthcare) using 10 mm sodium phosphate, 150 mm sodium chloride, pH 7.2, as the mobile phase. The top of the major peak was collected and loaded onto a biotin-Sepharose to remove trace of avidin. Unbound fraction was concentrated, filtered (0.22μ) and stored at −20 °C. Protein concentration was determined by Protein Dc Assay (Bio-Rad) using bovine serum albumin as the standard. Purity of OVAX was estimated by quantitative enumeration of pixels of the 45–50 kDa band relative to pixels of the whole lane (LI-COR Odyssey, LI-COR Biosciences, Inc., Lincoln, NE).

To better appreciate the affinity of OVAX for heparin, purified OVAX was injected on Hi-Trap heparin HP (GE Healthcare) equilibrated with 50 mm Tris, pH 7.4. OVAX was eluted with a NaCl linear gradient using 50 mm Tris, 1.5 m NaCl, pH 7.4, at 0.5 ml/min.

##### Identification of OVAX by Mass Spectrometry

The major protein band corresponding to 45 kDa was excised, rinsed, reduced, alkylated, and digested with Sequencing Grade Trypsin (Roche Diagnostics GmbH, Mannheim, Germany) as described by Shevchenko *et al.* (17). The tryptic fragments were extracted and dried. Several mass spectrometry approaches, MALDI and nanoESI mass fingerprint with sequencing by nanoscale capillary liquid chromatography-tandem mass spectrometry (nanoLC-MS/MS), were used to obtain the protein identification with the better sequence coverage.

NanoLC-MS/MS analysis of the digested peptides was performed using CapLC system coupled to a hybrid quadrupole time-of-flight mass spectrometer (UltimaGlobal, Waters, Manchester, UK) as described by Belleannee *et al.* (18). The nanoESI mass fingerprint was collected from the nanoLC-MS/MS analysis performed for the identification. All MS survey scans used for MS/MS mode, over a mass range of 400–1300 *m*/*z*, were combined from the chromatogram. MassLynx software (version 4.0, Waters, Manchester, UK) was used to process the spectrum by a background substract using a polynomial order of 10% below the curve and a smoothing step with the minimum peak width at half-height set to three channels. Two smoothing steps were performed using the Savitzky Golay algorithm. To obtain [M + H]^+^ masses from multicharged nanoESI mass spectrum, automatic deconvolution was applied with the MaxEnt 3 option. MALDI-TOF mass fingerprints were obtained with M@LDI-TOF L/R mass spectrometer (Waters, Manchester, UK) as described by Baranger *et al.* (19).

All molecular species (102 *m*/*z*) obtained with MALDI and nanoESI were matched automatically to proteins in a non-redundant database using MASCOT software (Matrix Science) against the Chordata section. Enzyme specificity was set to trypsin with four missed cleavages using carbamidomethylcysteine and methionine oxidation as variable modifications. The tolerance of the ions was set to 100 ppm. Protein hits were validated if the protein score was above the MASCOT default significance threshold (*p* < 0.05).

##### Biochemical Analysis of OVAX and Ovalbumin

Four μg of OVAX and S-free ovalbumin (Sigma-Aldrich, Saint-Quentin Fallavier, France) were diluted in sample buffer with or without β-mercaptoethanol. Samples with β-mercaptoethanol were boiled, and all samples were further analyzed by SDS-PAGE. Deglycosylation of OVAX (10.2 μg) and ovalbumin (10 μg) by PNGase F was performed following manufacturer's instructions.

##### Identification of Glycosylation Sites in OVAX Protein

Native OVAX and OVAX deglycosylated by PNGase F were separated by SDS-PAGE under reducing conditions, stained by Coomassie Blue, and in-gel digested by trypsin. Peptide mixtures were analyzed by online nanoflow liquid chromatography tandem high resolution mass spectrometry. All experiments were performed on a dual linear ion trap Fourier transform mass spectrometer LTQ Orbitrap Velos (Thermo Fisher Scientific) coupled to an Ultimate® 3000 RSLC chromatographer (Dionex, Amsterdam, The Netherlands) controlled by Chromeleon Software (Dionex). Five μl of each sample were injected using μl pickup mode and loaded on an LCPackings trap column. Mobile phases consisted of the following: 0.1% formic acid, 97.9% water, 2% acetonitrile (v/v/v) (A) and 0.1% formic acid, 15.9% water, 84% acetonitrile (v/v/v) (B). The gradient consisted of 4–55% B for 60 min, 55 to 99% B for 1 min, constant 99% B for 20 min, and a return to 4% B in 1 min at 300 nl/min. The eluate was nano-electrosprayed through a Thermo Finnigan Nanospray Ion Source 1 with a SilicaTip emitter of 15-μm inner diameter (New Objective, Woburn, MA). Standard mass spectrometric conditions for all experiments were as follows: spray voltage, 1.2 kV; no sheath and auxiliary gas flow; heated capillary temperature, 250 °C; predictive automatic gain control enabled; and an S-lens RF level of 60%.

Data were acquired using Xcalibur software (Thermo Fisher Scientific). The LTQ Orbitrap Velos instrument was operated in positive mode in a data-dependent mode to automatically switch between full scan Fourier Transform-MS and Fourier Transform-MS/MS spectra. Resolution in the Orbitrap was set to *r* = 60,000. In the scan range of *m*/*z* 300–1800, the 10 most intense peptide ions with charge states ≥2 were sequentially isolated and fragmented by higher-energy collisional dissociation (normalized collision energy of 40%). Dynamic exclusion was activated during 30 s with a repeat count of 1. Polydimethyl-cyclosiloxane (*m/z* of 445.1200025) ions were used for internal calibration. Raw data files were converted with Proteome Discoverer software (version 1.3; Thermo Fischer Scientific). Precursor mass range of 350–5000 Da and signal-to-noise ratio of 1.5 were the criteria used for generation of peak lists. To characterize the glycosylated peptides, the data obtained were matched automatically against a locally maintained house database with sequences of interest and contaminants (516 sequences). MS/MS ion searches were performed using MASCOT software as described above. The tolerance of the ions was set to 5 ppm for parent and 0.1 Da for fragment ion matches, and the instrument setting was specified as “ESI-Fourier transform ion cyclotron resonance mass spectrometry.” Peptide hits were validated when an expected score was < 0.05.

##### Enzymatic Assays

Assays were carried out in 96-well microplates using a Tecan Infinite M200 microplate reader (Tecan France, Lyon, France). Antitrypsin activity of OVAX was assessed by incubating trypsin (5 nm), OVAX (0–500 nm) with or without heparin (10, 100 μg/ml final concentration), or α1-antitrypsin (0–500 nm) in 50 mm Hepes, 0.1 m NaCl, pH 8, for 1 h at 37 °C prior to addition of tosyl-GPR-pNA substrate (0.3 mm). The slope obtained after a 20 min was compared with that of the control consisting of trypsin (5 nm) and tosyl-GPR-pNA (0.3 mm). Anti-chymotrypsin activity was studied as described above except that we used cathepsin G as the enzyme (95 nm), OVAX or α1-antichymotrypsin (0–500 nm), and Suc-AAPF-pNA (0.3 mm) substrate. Inhibitory activity of α2-antiplasmin (0–90 nm) and OVAX (0–190 nm) against plasmin (16 nm) was assessed after a 1-h incubation at 37 °C using VLK-pNA (0.3 mm) in 50 mm Hepes, 0.1 m NaCl, pH 8.

##### CD Spectroscopy

Estimation of secondary structure content and thermal denaturation were assessed by far-UV circular dichroism. For secondary structure analysis, CD spectra were recorded on a Jasco J-810 spectropolarimeter from 260 to 190 nm using a 1-mm jacketed quartz cell at 20 °C, a scan rate of 20 nm/min, a response time of 8 ms, a bandwidth of 1 nm, a resolution of 1 nm, and a protein concentration of 0.1 mg/ml. Each spectrum represents the average of three scans, with the buffer subtracted. The estimation of secondary structure content from circular dichroism measurements was performed using the CDPro software package (reference protein set selected, SPD48). Regarding thermal denaturation, the unfolding transitions curves were obtained by measuring the ellipticity at 222 nm every 0.2 °C in a 1-mm cell. The temperature was raised from 20 to 90 °C with a 2 °C/min gradient. The denaturation temperature of unfolding (mid-transition temperature) was determined assuming that the unfolding equilibrium followed a two-state mechanism.

##### OVAX Homology Modeling

Automated homology modeling was performed by using the SWISS-MODEL workspace (20). All of the modeling parameters were set to the default. The OVAX three-dimensional model was built based on the 1.95 Å resolution structure of chicken uncleaved ovalbumin (Protein Data Bank code 1OVA) as a specific template.

##### Antimicrobial Tests

Antibacterial activities of OVAX, ovalbumin, and avian β-defensin-11 (positive control (12)) were investigated against *Listeria monocytogenes* EGD strain (*L. monocytogenes*), *Salmonella enterica* sv. Enteritidis ATCC 13076 (*S. enterica* sv. Enteritidis), *Escherichia coli* ATCC 25922 (*E. coli*), and *Staphylococcus aureus* ATCC 29740 (*S. aureus*) as described previously (12). Antimicrobial activities of avian β-defensin 11 (10–100 μg/ml, 9271.56 Da (12)), ovalbumin (0.5–2.2 mg/ml, 42.9 kDa), and OVAX (0.5–2.2 mg/ml, 45,430 kDa) were assessed using radial diffusion assay (12, 21). The minimum active concentration (22) of each molecule was calculated by linear regression to determine the minimal concentration of molecules required to observe a measurable clear zone (0.5 mm). The effect of heparin (10, 50, 100 and 1000 μg/ml) was evaluated using 1.2 mg/ml and 0.2 mg/ml OVAX for *S. enterica* sv. Enteritidis and *L. monocytogenes*, respectively.

## RESULTS

### 

#### 

##### Tissue Distribution of OVAX

**FIGURE 1.**
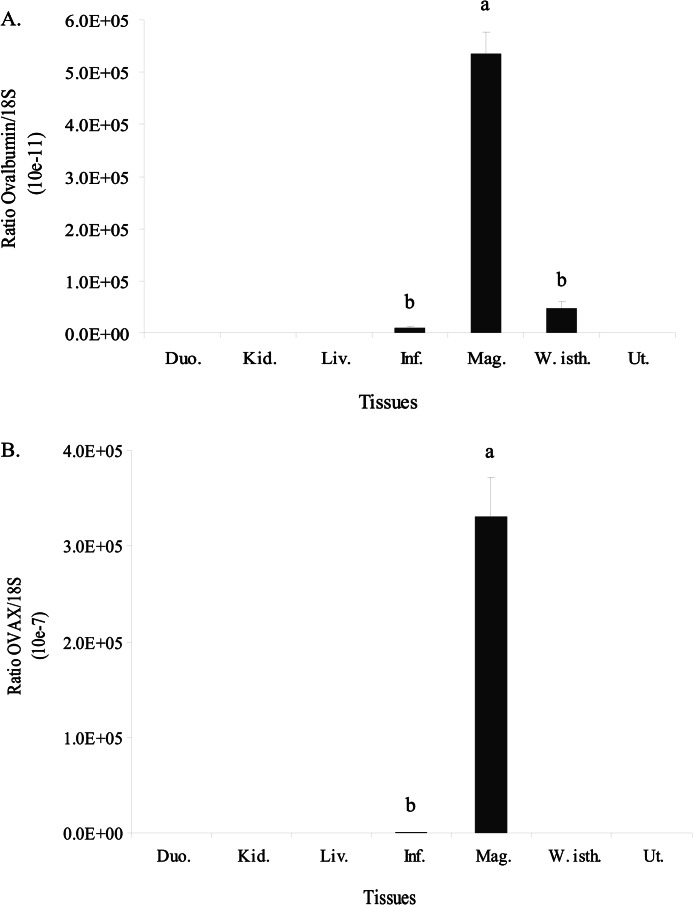
**Tissue expression of ovalbumin (*A*) and OVAX (*B*) in chicken hens.** Means that do not share a common *letter* are significantly different (*p* < 0.05; *n* = 8). *Duo.*, duodenum; *Kid.*, kidney; *Liv.*, liver; *Inf.*, infundibulum; *Mag.*, magnum; *W. Isth.*, white isthmus; *Ut.*, uterus. Ratio are expressed as arbitrary units.

Real-time RT-PCR performed with specific primers to ovalbumin and to OVAX (Fig. 1, *A* and *B*, respectively) revealed that the expression of OVAX is highly similar to that of ovalbumin: OVAX is specifically expressed by the magnum (*p* < 0.0001), which is responsible for egg white formation. No expression was observed in duodenum, kidney, or liver (involved in egg yolk formation), whereas the expression in other oviduct tissues, including infundibulum (formation of vitelline membranes of chicken egg), white isthmus (formation of eggshell membranes), or uterus (formation of eggshell) was shown to be negligible compared with expression in magnum.

##### Purification of OVAX from Egg White

**FIGURE 2.**
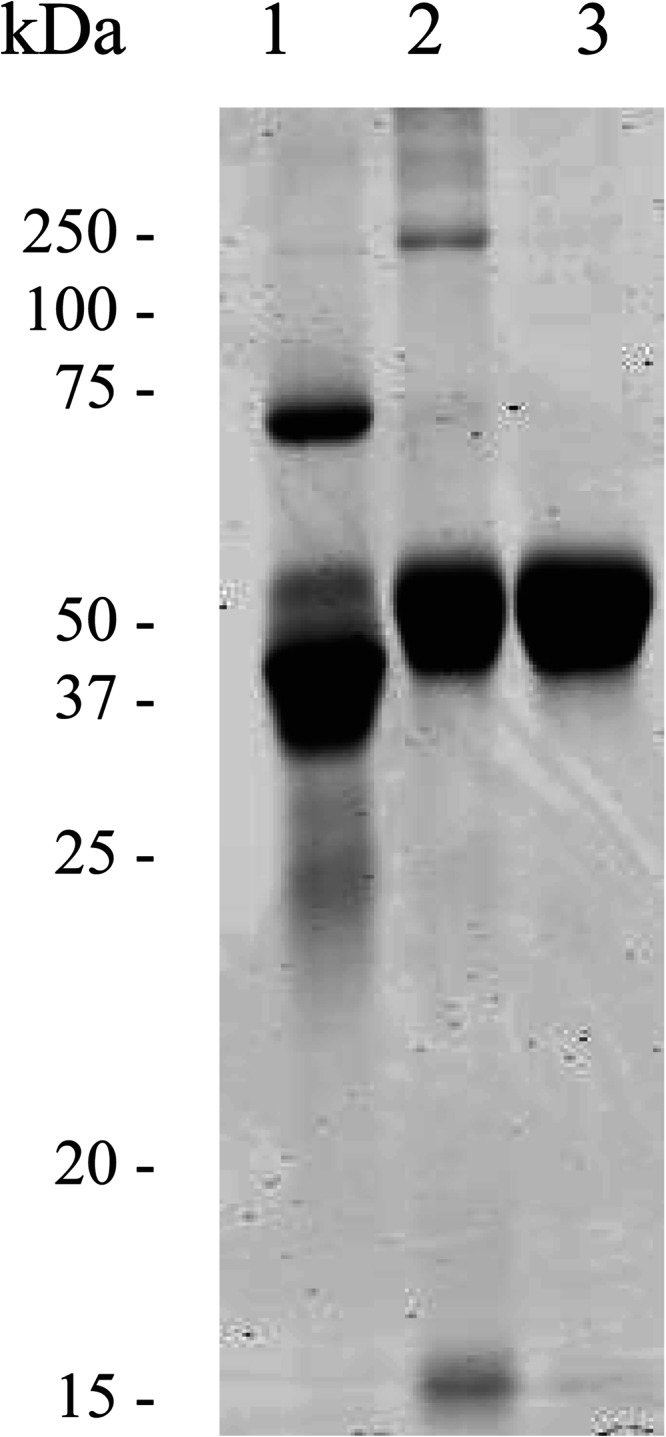
**Purification of a heparin-binding protein from egg white.** *Lane 1*, egg white; *lane 2*, heparin-binding proteins from egg white; *lane 3*, purified OVAX. Samples were analyzed by SDS-PAGE followed by Coomassie Blue staining.

As shown by SDS-PAGE under non-reducing conditions (Fig. 2), the heparin-bound fraction of egg white contains a major band showing an apparent molecular mass of 45–50 kDa (Fig. 2, *lane 2*), which was further purified. The fraction was submitted to gel filtration, and the top of the major peak was passed through biotin-Sepharose to remove avidin, which is a known heparin-binding molecule (23) and is co-purified with OVAX as a tetramer of ∼45 kDa. The purity of resulting protein was estimated to be 98% by densitometry (Fig. 2, *lane 3*; “Experimental Procedures”). Eight mg of pure protein at least can be recovered from 30 ml of egg white, which corresponds to ∼0.3 mg per ml of egg white. This concentration is likely to be underestimated because multimers as well as fractions containing other protein contaminants were discarded as a result of gel filtration.

##### Identification by Mass Spectrometry

The SDS-PAGE 45-50-kDa band was analyzed by mass spectrometry after tryptic digestion. Combination of MALDI and nanoESI mass fingerprints with sequence information from nanoLC-MS/MS analysis allowed for the identification of a protein related to OVAY (predicted sequence, ovalbumin-related protein Y (XP_418984.3; GI:118086485)) with 85% coverage (data not shown). The NCBI sequence of this protein has been predicted by automated computational analysis using the NCBI gene prediction method (Gnomon). Three different versions of the protein have been updated since 2004. These three predicted sequences differ mainly in their amino-terminal extremity. The XP_418984.3 version predicts the presence of nine additional residues at the amino-terminal of the sequence. These residues (MFFYNTDFR) were further confirmed by mass spectrometry analyses (data not shown). Several authors have shown that this protein (predicted sequence: ovalbumin-related protein Y) is actually ovalbumin-related protein X (24, 25) or OVAX.

##### PCR Amplification of OVAX Transcript

**FIGURE 3.**
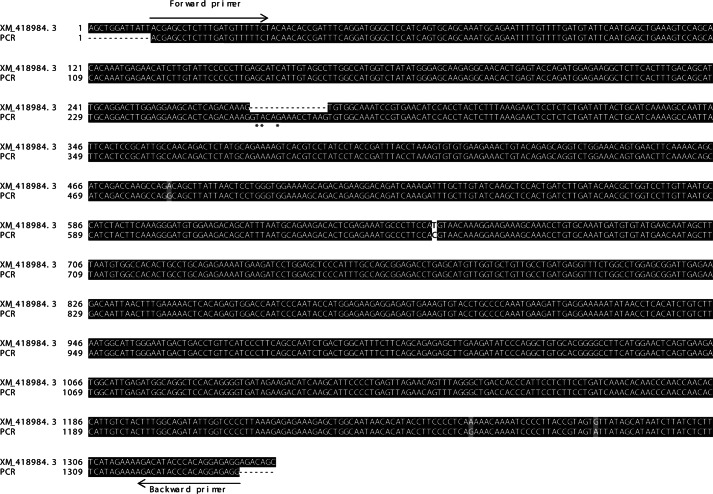
**Alignment between OVAX transcript XM_418984.3 and OVAX PCR product.** *Arrows* indicate starting and stop codons. Identical residues are shaded *black*, and mutations appear in *gray* or *white. Asterisks* indicate sequencing ambiguities. Alignments were performed using CLUSTALW and BOXSHADE software.

To further confirm the predicted sequence published by NCBI, we amplified OVAX transcript from magnum tissue. Sequencing of the PCR product of OVAX transcript revealed the presence of additional nucleotides GTACAGAAACCTAAG corresponding to a VQKPK residues (Fig. 3). Although some ambiguities were detected on three of the corresponding 15 nucleotides (illustrated by *asterisks* on Fig. 3), T/G, A/C, T/C/G, after six runs of sequencing, the sequence GTACAGAAACCTAAG was found to be present in the OVAX gene (gi:358485510). Interestingly, the prediction of OVAX protein sequence performed using the GeneMark tool as opposed to Gnomon (NCBI) using the gene sequence available at the NCBI server (GeneID 420898, NC_00689.3, released on February 27, 2012) also gives this additional VQKPK peptide (supplemental Fig. 2). Additionally, two noncausal mutations were reported on nucleotide 484 (AGA, AGG coding arginine) and on nucleotide 666 (CAT, CAC coding histidine) of the coding sequence (Fig. 3). Two other mutations were detected out of the coding sequence (nucleotides 1259 A/G and 1283 G/A).

##### Biochemical Properties of OVAX

**FIGURE 4.**
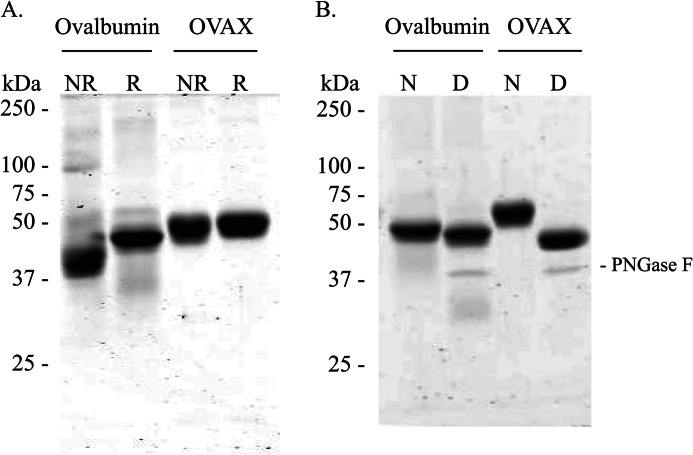
**Comparison of physicochemical features between ovalbumin and OVAX.** *A*, electrophoretic mobility of ovalbumin and OVAX under non-reducing/non-boiling conditions (*NR*) and reduced/boiled (*R*) conditions. *B*, analysis of ovalbumin and OVAX glycosylations. *N*, native; *D*, after deglycosylation using PNGase F.

**FIGURE 5.**
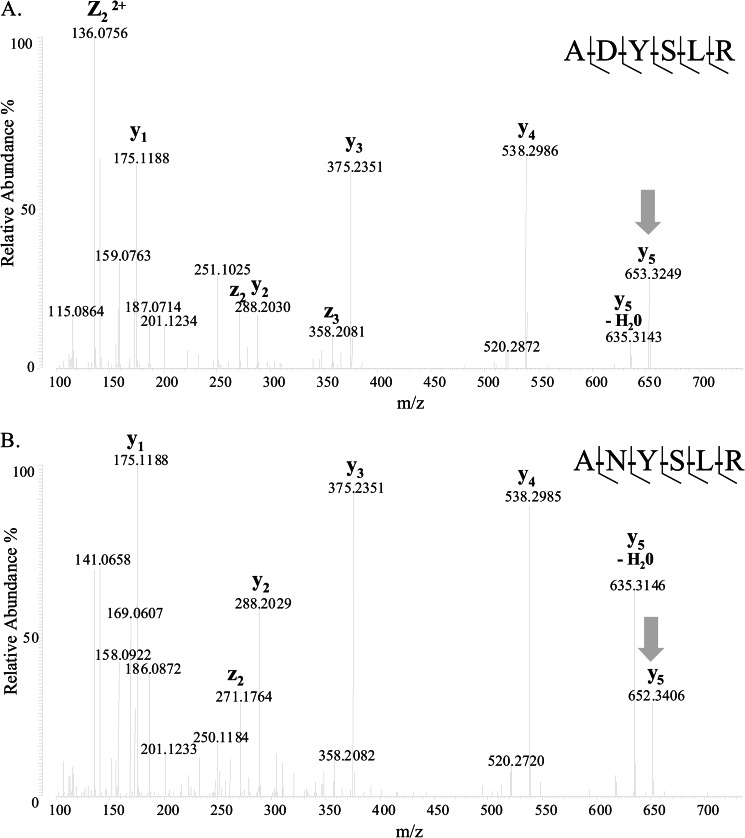
**Heterogeneity of Asn^102^ glycosylation site.** *A*, MS/MS spectrum assigned to the deglycosylated OVAX peptide. The bicharged precursor ion (*m*/*z*) 362.6849 ([M + H]^+^ = 724,3551) was isolated and fragmented by higher-energy collisional dissociation. Fragment ions containing the C terminus (*y*- and *z*-type ions) are labeled. The characterization of the *N*-deamidation (+0.98402) by the observed *y*_5_ fragment ion (*m*/*z* 653,3249; *gray arrow*) confirmed the conversion of asparagine to aspartate by the PNGase F and therefore the site of glycosylation on Asn^102^. *B*, MS/MS spectrum assigned to non-glycosylated ANYSLR peptide (bicharged precursor ion (*m*/*z*) 362,1930, [M + H]^+^ = 723,386) showing post-translational modifications heterogeneity within OVAX.

Injection of OVAX onto a hi-Trap Heparin HP column using a linear gradient of NaCl reveals that OVAX has high affinity for heparin because its elution from the column was initiated with 720 mm NaCl (data not shown). SDS-PAGE analysis of OVAX and ovalbumin under non-reducing/non-heat-denaturing conditions revealed some diverging electrophoretic profiles. Ovalbumin gives an apparent molecular mass of 38–40 kDa under SDS non-denaturing conditions (Fig. 4*A*, *lane 1*), whereas it shows an increase in weight under reducing conditions (Fig. 4*A*, *lane 2*). In contrast, the electrophoretic profile of OVAX seems to be not altered by one or the other condition (Fig. 4*A*, *lanes 3* and *4*). We also examined post-translational glycosylations of OVAX compared with ovalbumin knowing that the latter is reported to be monoglycosylated (26). We observed a higher shift of apparent molecular mass between the native and deglycosylated form of OVAX (Fig. 4*B*, *lanes 3* and *4*) compared with ovalbumin (Fig. 4*B*, *lanes 1* and *2*). These data suggest that OVAX is a glycoprotein. Investigation of glycosylations using Center for Biological Sequence analysis server prediction (NetNGlyc server (version 1.0)) revealed that OVAX contains five potential *N*-glycosylated sites N102YS, N220NS, N298LT, N317LT, N379PT. Two of these sites, N102YS and N220NS, were further corroborated by nanoLC-High resolution MS/MS analysis. As shown for N102YS, asparagine was converted to aspartate after PNGase F treatment (Δmass, +0.98402 Da; Fig. 5*A*). However, this site was also identified as non-glycosylated (Fig. 5*B*). A similar observation was made for the site N220NS. These results suggest high glycosylation heterogeneity for OVAX protein and are consistent with the electrophoretic profile of OVAX showing a large band on SDS-PAGE. The predicted site N379PT was shown to be non-glycosylated, whereas no information related to sites N298LT and N317LT was obtained, as the corresponding peptides were not selected for fragmentation.

##### Assessment of the Inhibitory Activity of OVAX against Proteases

**FIGURE 6.**
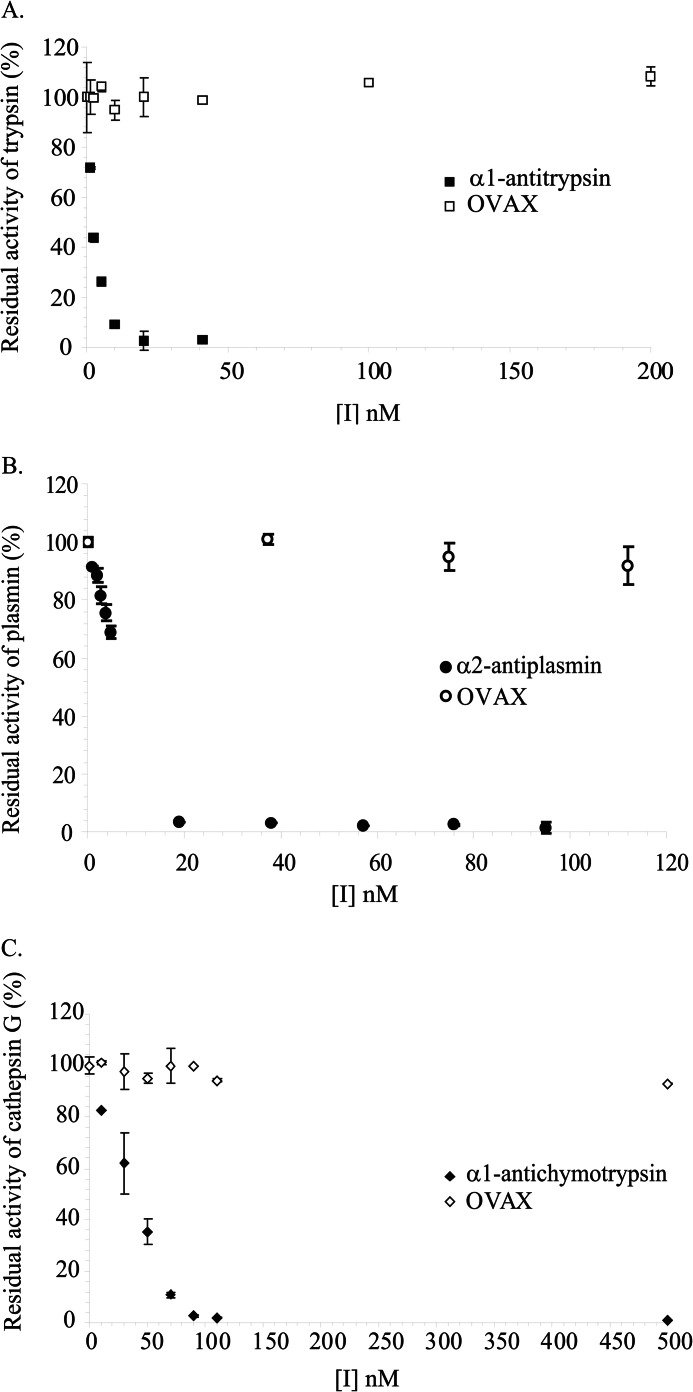
**Inhibition of trypsin (*A*), plasmin (*B*), and cathepsin G (*C*) by OVAX and inhibitory serpins.** Reactions were performed as described under “Experimental Procedures.” Results express the average of at least two independent experiments done in duplicate. *I*, inhibitor.

Kinetic assays with chromogenic substrates (Fig. 6) revealed that OVAX was not able to inhibit trypsin or plasmin (trypsin-like proteases) and cathepsin G (chymotrypsin-like protease), although all of these were inhibited by their respective inhibitory serpins (α1-antitrypsin, α2-antiplasmin, α1-antichymotrypsin). The same results were obtained when using heparin (10 μg/ml or 100 μg/ml) as a potential cofactor (data not shown).

##### Far-UV CD Analysis of OVAX and Ovalbumin

**FIGURE 7.**
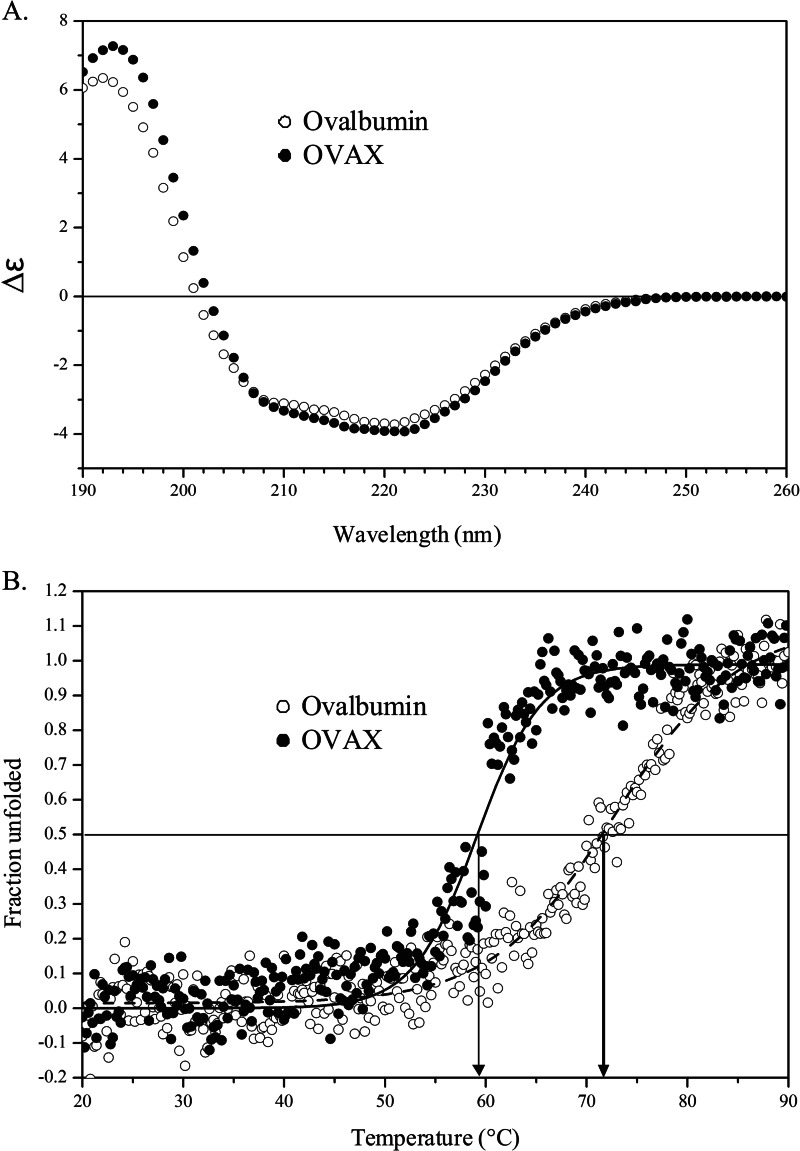
**Circular dichroism analyses of OVAX and ovalbumin proteins.** *A*, far-UV CD spectra of OVAX (*filled circles*) and ovalbumin (*empty circles*). The unit on the ordinate is mean differential absorption coefficient (Δϵ). *B*, thermal denaturation of OVAX (*filled circles*) and ovalbumin (*empty circles*) monitored by following the changes in ellipticity at 222 nm.

The far-UV CD spectra of ovalbumin and OVAX were very similar and showed the typical signature of α/β class of proteins with minima at 220 and 208 nm. As expected from the spectra, the secondary structures of OVAX and ovalbumin are highly similar (Fig. 7*A*) with an α-helical content of 34% and a β-sheet content of 16% for ovalbumin, whereas OVAX had 34% α-helix and 19% β-sheet.

Spectra recorded at different temperatures revealed one isodichroic point at ∼205 nm for ovalbumin and OVAX (data not shown), indicating a simple two-state mechanism for unfolding process. According to the thermal denaturation CD spectra, the *T_m_* values of OVAX and ovalbumin were 58.9 and 71.2 °C, respectively (Fig. 7*B*).

##### Three-dimensional Model of OVAX

**FIGURE 8.**
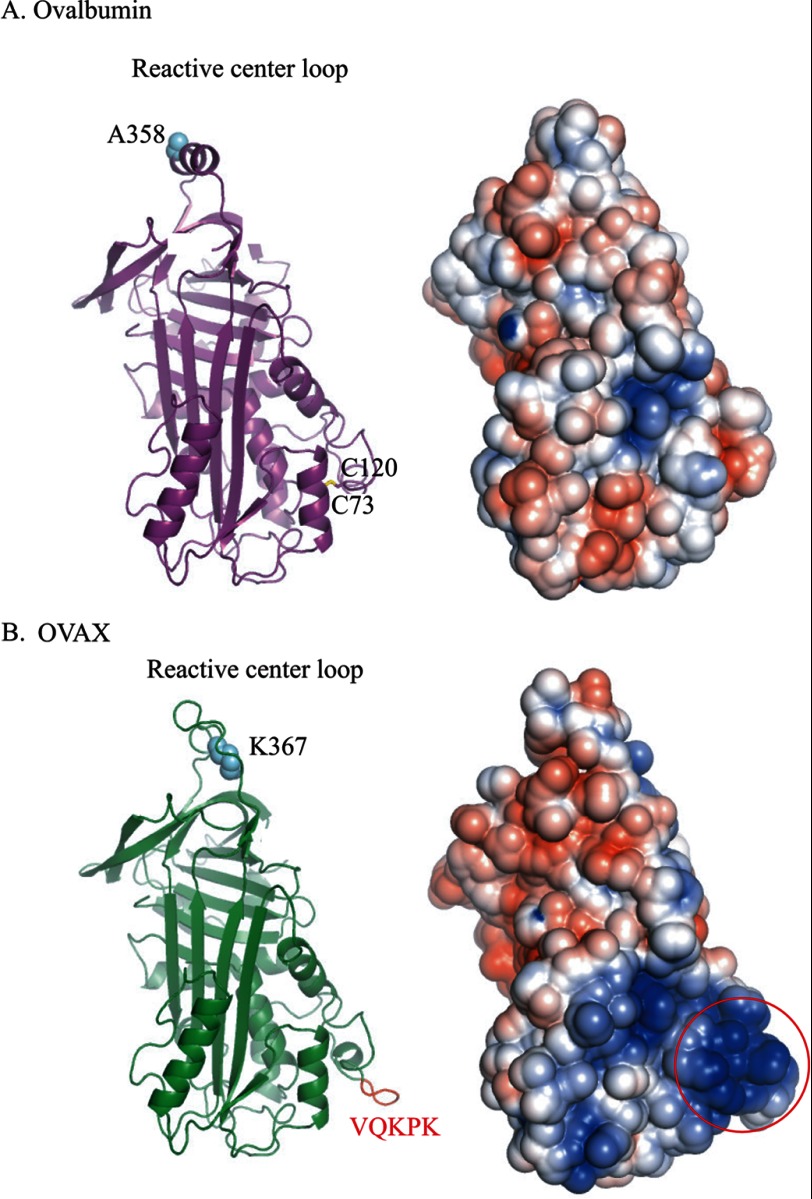
**Modeling of OVAX from the three-dimensional structure of ovalbumin.** *A*, ovalbumin. *Left panel*, schematic representation of the three-dimensional structure of ovalbumin (*purple*). The disulfide bond between Cys^73^ and Cys^120^ is indicated in *yellow. Right panel*, electrostatic potentials mapped over the molecular surface of ovalbumin. *B*, OVAX. *Left panel*, schematic representation of the three-dimensional structure model of OVAX (*green*). The OVAX pentapeptide VQKPK is highlighted in *red. Right panel*, electrostatic potentials mapped over the molecular surface of OVAX. The *red circle* highlights the putative heparin binding area. P1 reactive sites are Ala^358^ for ovalbumin and Lys^367^ for OVAX.

A three-dimensional structure of OVAX was modeled by homology to the x-ray structure of uncleaved ovalbumin (Protein Data Bank code 1OVA). As amino-terminal residues of OVAX (MFFYNTDFR) were not present in the ovalbumin sequence, their modeling by homology was not possible. Therefore, the resulting model of OVAX corresponds to residues 11 to 402. Fig. 8 illustrates the ovalbumin structure (Fig. 8*A*, *left panel*, *purple*) containing the Cys^73^-Cys^120^ disulfide bond compared with the resulting OVAX model (Fig. 8*B*, *left panel*, *green*), including the pentapeptide VQKPK highlighted in *red*. The distribution of positive charges (*blue*) and negative charges (*red*) on the molecular surface of ovalbumin (Fig. 8*A*, *right panel*) and OVAX (Fig. 8*B*, *right panel*) reveals in OVAX structure, a cluster of positive charges corresponding to the following sequence: STQTKVQKPKCGKSVNIHLLFKELLSDITASKANYSLRIANRLYAEKSRPILPIYLKCVKK. This domain, which includes the VQKPK peptide identified by PCR amplification (see above), forms a bulky domain that is pointed out toward the surface of the molecule. This exposed domain is likely to be involved in the interaction with negatively charged surfaces, including heparin glycosaminoglycan.

##### Assessment of the Antimicrobial Potential of OVAX

**FIGURE 9.**
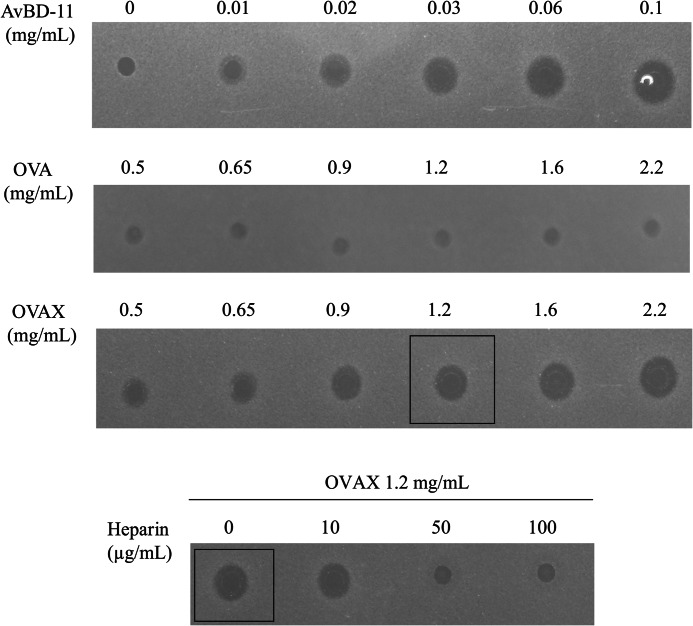
***S. enterica* sv. Enteritidis growth inhibition test using radial diffusion assay.** The clearance zones correspond to the inhibitory effect of avian β-defensin 11(AvBD11), ovalbumin, and OVAX at various concentrations. Heparin effect was assessed using 1.2 mg/ml of OVAX and 10, 50, or 100 μg/μl of heparin. Controls for heparin (10 and 100 μg/ml) and buffer (Tris-buffered saline) exhibited no inhibition (data not shown).

Antimicrobial potential of OVAX and ovalbumin was assessed against *S. enterica* sv. Enteritidis and *E. coli* (Gram-negative strains), and *L. monocytogenes* and *S. aureus* (Gram-positive strains). Avian β-defensin 11, an egg white antimicrobial peptide (12) was used as a positive control. Results are presented in Table 1. We showed that OVAX, unlike ovalbumin, was able to inhibit *S. enterica* sv. Enteritidis (Table 1 and Fig. 9) and *L. monocytogenes* (Table 1). Interestingly, we also demonstrated that heparin was able to inhibit the anti-*S. enterica* sv. Enteritidis activity of OVAX at concentrations as low as 50 μg/ml (Fig. 9). In contrast, no significant effect of heparin was observed on the anti-*L. monocytogenes* potential of OVAX (data not shown).

**TABLE 1:** **Minimal active concentration of purified OVAX, ovalbumin, and av-BD11 (positive control)** MAC indicates minimum active concentration (corresponding to a 0.5-mm clear zone). Gram+, Gram positive; Gram−, Gram negative.

Bacterial group/strains	MAC (μm) ± S.D.		
	AvBD11	Ovalbumin	OVAX
*L. monocytogenes* (Gram+)	0.90 ± 0.83	>28	1.90 ± 0.49
*S. aureus* ATTCC 29740 (Gram+)	7.73	>37	>37
*S. enterica* sv. Enteritidis ATCC 13076 (Gram−)	0.79 ± 0.49	>51	10.02 ± 1.36
*E. coli* ATCC 25922 (Gram−)	1.6	>37	>37

## DISCUSSION

Ovalbumin is the major protein of egg white and is believed to constitute a nutrient for the developing chicken embryo. During embryogenesis, ovalbumin undergoes a thermal change to form a more stable structure named as S-ovalbumin, while migrating to amniotic fluid, embryonic organs, and yolk (27). Ovalbumin is not capable of protease inhibition by the “serpin mechanism,” and little is known regarding its putative functions. The two related proteins OVAY and OVAX have been described. Although the biochemical characteristics of OVAY have been published recently (28, 29), there are, to date, no data regarding OVAX and its related function.

We first explored the specificity of expression of OVAX compared with that of ovalbumin to identify the most relevant egg source of OVAX. Results revealed that OVAX is specifically expressed by the magnum, considering that only a slight expression was observed in the other oviduct tissues (infundibulum, isthmus, and uterus). This pattern of expression is consistent with its preferred localization in egg white, similar to ovalbumin (24, 30, 31). However, OVAX is believed to be a 100 times less concentrated than ovalbumin in egg white based on expression (4). The differential responsiveness of OVAX and OVAY genes compared with ovalbumin gene is supposed to contribute to their differential transcription rates and to the markedly different steady-state levels of their respective gene transcripts (4). It has been shown that the synthesis of egg white proteins depends on levels and types of steroid hormones but also on the combination of the two (32). Assuming that the concentration of ovalbumin is ∼50 mg/ml egg white, we estimated the concentration of OVAX to be 0.5 mg/ml in egg white. The yield of purification of OVAX using heparin-Sepharose followed by gel filtration and biotin-Sepharose gave a concentration of ∼0.3 mg of OVAX per ml of egg white, which is in the same range as the estimated value (4).

Although highly related in sequences (nucleic and amino acid sequences (1, 2) and supplemental Fig. 1), we showed that ovalbumin and OVAX possess specific biochemical and biophysical features: 1) OVAX exhibits a high affinity for heparin, in contrast to ovalbumin; 2) OVAX is assumed to have multiple glycosylation sites (two of them were confirmed) and exists as multiple glycoforms, whereas ovalbumin is monoglycosylated on Asn^292^ (33); and 3) a higher electrophoretic mobility was observed for ovalbumin as compared with OVAX under moderately denaturing conditions but not under reducing conditions. We also showed that OVAX possesses a lower *T_m_* compared with that of ovalbumin (58.9 °C for OVAX *versus* 71.2 °C for ovalbumin), which suggests that OVAX might be less stable than ovalbumin.

OVAX belongs to the serine protease inhibitor (serpin) family with a predicted reactive site at Lys^367^-His^368^. No inhibitory activity against trypsin-like proteases or against two chymotrypsin-like proteases, cathepsin G and chymotrypsin (data not shown), could be detected, even using heparin as a potential cofactor, as reported for other serpins (34–37). Serpins are known inhibitors of serine proteases, but some members of this family, such as squamous cell carcinoma antigen 1, inhibit cysteine proteases (38). No inhibition of the cysteine protease papain by OVAX could be detected (data not shown). To conclude, we could not find any evidence of inhibitory activity of OVAX, considering the several proteases tested.

As mentioned above, the main feature characterizing OVAX as compared with ovalbumin is its ability to bind heparin, a negatively charged glycosaminoglycan. Consensus binding sites to heparin have been defined, consisting of XBBXBX or XBBBXXBX where B is an arginine or a lysine and X a hydropathic residue (39). Both OVAX and ovalbumin possess such predicted site(s): ERKIKV (277–280) for ovalbumin and KRRVKV (290–294) for OVAX. From these analyses, both proteins could potentially bind to heparin, although we found that only OVAX had affinity for this glycosaminoglycan. However, several articles (40, 41) suggest that the binding of proteins to heparin requires several basic amino acids, which are not necessarily adjacent in the primary sequence, but rather are in spatial proximity. We therefore searched for such positively charged domains that could potentially explain the difference in binding to heparin between these two related proteins. Considering that ovalbumin and OVAX have similar content in secondary structures and that they are highly similar in sequence, we modeled OVAX based on the three-dimensional structure of uncleaved ovalbumin. We showed that OVAX, in contrast to ovalbumin, displays a specific exposed domain with a high content of positive residues grouped together at the surface of the molecule. This domain includes the pentapeptide VQKPK, which was not predicted in the various public sequences but that we could prove by PCR amplification of the corresponding transcript and by internal Edman sequencing (data not shown). Additional studies will be initiated in future to investigate the three-dimensional structure of OVAX and to further identify the precise sequence of its heparin-binding site(s).

There is increasing evidence in literature that heparin-binding proteins and peptides participate in host defense against pathogens (11–14). According to Andersson *et al.* (11), the requirements for heparin interaction with molecules (including amphipaticity and cationicity) are strikingly similar to the structural features of known antimicrobial peptides. We therefore explored the antimicrobial potential of OVAX and demonstrated that this egg white serpin with heparin affinity is antimicrobial against both Gram-negative and Gram-positive bacteria. Moreover, we showed that the antimicrobial activity of OVAX against *Salmonella* could be abolished by co-incubation of OVAX with purified heparin. The fact that heparin interferes with the anti-*Salmonella* but not the anti-*Listeria* activity of OVAX suggests that the mechanisms underlying the antimicrobial activity of OVAX, require multiple domains, and suppose different modes of action. Additional studies using heparin-binding site variants of OVAX will be needed to further confirm the involvement of heparin-binding site(s) in the anti-*Salmonella* activity. It might also be interesting in future to assess whether a peptide spanning the heparin binding site(s) of OVAX would have similar (or increased) activity, as it has been reported for heparin-binding protein C inhibitor (13) and other peptides derived from heparin-binding proteins (11).

Our result demonstrate for the first time that OVAX contribute to egg defense against pathogens, to protect the embryo against microbial contamination, and during egg storage, to maintain the food safety of the table egg. Beyond its interest as a new natural antimicrobial agent with a specific and peculiar mode of action, such molecule could have potential industrial uses as food conservatives or as pharmaceuticals (42), similarly to lysozyme, a major egg white antimicrobial protein (43, 44).

## Footnotes

2 The abbreviations used are:

OVAY: ovalbumin-related protein Y
nanoESI: nanoelectrospray ionization
PNGase F: peptide-*N*-glycosidase F
OVA: ovalbumin
OVAX: ovalbumin-related protein X
ov-serpin: ovalbumin-serpin
nanoLC-MS/MS: nanoscale capillary liquid chromatography-tandem mass spectrometry.
